# TAM-ing the CIA—Tumor-Associated Macrophages and Their Potential Role in Unintended Side Effects of Therapeutics for Cancer-Induced Anemia

**DOI:** 10.3389/fonc.2021.627223

**Published:** 2021-03-25

**Authors:** Stefan Weiler, Manfred Nairz

**Affiliations:** ^1^ National Poisons Information Centre, Tox Info Suisse, Associated Institute of the University of Zurich, Zurich, Switzerland; ^2^ Department of Chemistry and Applied Biosciences, Institute of Pharmaceutical Sciences, Eidgenossische Technische Hochschule Zurich, Zurich, Switzerland; ^3^ Department of Internal Medicine II, Infectious Diseases, Immunology, Rheumatology, Pneumology, Medical University of Innsbruck, Innsbruck, Austria

**Keywords:** cancer-induced anemia (CIA), tumor-associated macrophage (TAM), iron, hepcidin, ferroportin, BMP - Smad signaling pathway, IL-6 (interleukin 6)

## Abstract

Cancer-induced anemia (CIA) is a common consequence of neoplasia and has a multifactorial pathophysiology. The immune response and tumor treatment, both intended to primarily target malignant cells, also affect erythropoiesis in the bone marrow. In parallel, immune activation inevitably induces the iron-regulatory hormone hepcidin to direct iron fluxes away from erythroid progenitors and into compartments of the mononuclear phagocyte system. Moreover, many inflammatory mediators inhibit the synthesis of erythropoietin, which is essential for stimulation and differentiation of erythroid progenitor cells to mature cells ready for release into the blood stream. These pathophysiological hallmarks of CIA imply that the bone marrow is not only deprived of iron as nutrient but also of erythropoietin as central growth factor for erythropoiesis. Tumor-associated macrophages (TAM) are present in the tumor microenvironment and display altered immune and iron phenotypes. On the one hand, their functions are altered by adjacent tumor cells so that they promote rather than inhibit the growth of malignant cells. As consequences, TAM may deliver iron to tumor cells and produce reduced amounts of cytotoxic mediators. Furthermore, their ability to stimulate adaptive anti-tumor immune responses is severely compromised. On the other hand, TAM are potential off-targets of therapeutic interventions against CIA. Red blood cell transfusions, intravenous iron preparations, erythropoiesis-stimulating agents and novel treatment options for CIA may interfere with TAM function and thus exhibit secondary effects on the underlying malignancy. In this Hypothesis and Theory, we summarize the pathophysiological hallmarks, clinical implications and treatment strategies for CIA. Focusing on TAM, we speculate on the potential intended and unintended effects that therapeutic options for CIA may have on the innate immune response and, consequently, on the course of the underlying malignancy.

## Cancer-Induced Anemia Is a Frequent Consequence of Malignancy

Cancer-induced anemia (CIA) occurs in roughly one to two thirds of patients with solid tumors and complicates the course of malignancy (1–5). Its incidence is highly dependent on patient-related factors, on the entity and stage of the underlying disease and on therapeutic interventions. Specifically, the frequency and degree of anemia is higher in metastatic cancers, in aggressive hematologic malignancies and following treatment with high-dose chemotherapy, multi-targeted tyrosine kinase inhibitors and chimeric antigen receptor T cells (6–9). Therefore, CIA forms a spectrum which can broadly be categorized into three principal etiologies: First, CIA present before the initiation of anti-tumor therapy is typical of advanced disease stages with infiltration and replacement of the bone marrow or when the primary neoplasia results in substantial bleeding such as in colorectal or genitourinary malignancies. Second, indirect effects of products of neoplasms can lead to hemophagocytosis, autoantibody induced hemolysis or cytokine inhibition of erythropoiesis. Third, CIA with initial presentation only after the onset of anti-neoplastic treatment is one of the most common side effects of chemotherapy, yet also occurs as sign of progressive disease (10). According to the common terminology criteria for adverse events by the World Health Organization and National Cancer Institute, anemia is categorized into 5 grades from mild (Hemoglobin (Hb) 10 g/dL – lower limit of normal), to moderate (Hb 8.0 – 9.9 g/dL), severe (Hb <8 g/dL) and life-threatening with urgent interventions indicated (grade 4) or even death (grade 5) (11).

## Cancer-Induced Anemia Has Distinct Pathophysiological Hallmarks

**Figure 1:**
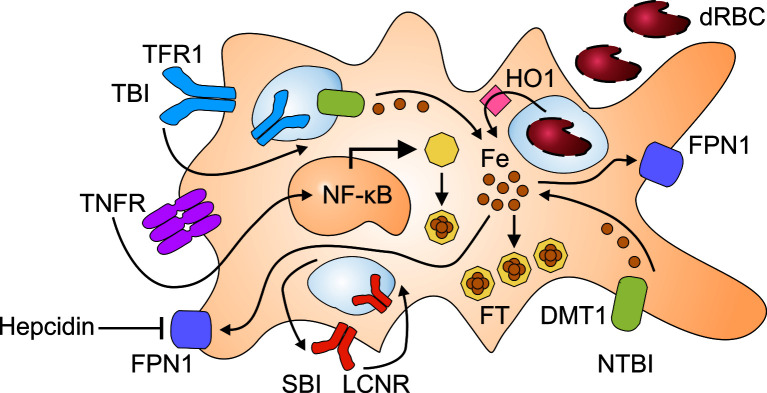
Pathways for the retention of iron in macrophages. Immune activation results in the production of inflammatory cytokines such as tumor necrosis factor (TNF) which damage red blood cells (dRBC) and activate nuclear factor (NF)-κB. The uptake of dRBC delivers large amounts of heme to macrophages which recycle heme-derived iron after its release by heme oxygenase (HO)-1. Moreover, inflammatory cytokines stimulate the uptake of transferrin-bound iron (TBI) *via* transferrin receptor (TFR)-1 and of non-transferrin-bound iron (NTBI) *via* divalent metal transporter (DMT)-1. TBI incorporated *via* TFR1 undergoes reduction to its ferrous form in the endosome and subsequent transfer to the cytoplasm through DMT1. The lipocalin-2 receptor (LCNR) can mediate both the uptake and the release of siderophore-bound iron (SBI). Inflammatory cytokines and hepcidin reduce ferroportin (FPN)-1 mediated iron export, which further contributes to iron retention in macrophages. To avoid elevated iron levels in the cytoplasm, labile iron is incorporated into ferritin (FT) which is upregulated both by iron and NF-κB.

The pathophysiology of CIA is complex and involves several contributing mechanisms. First, the immune response against malignant cells inevitably induces the iron-regulatory hormone hepcidin, which then directs iron fluxes away from the erythron and into compartments of the mononuclear phagocyte system (MPS) (**Figure 1**). In addition, certain inflammatory mediators, many of them cytokines such as tumor necrosis factor (TNF), interleukin (IL)-1, IL-6, transforming growth factor (TGF)-ß and IL-10, stimulate iron uptake into the MPS, induce iron storage in the form of ferritin (FT) and/or block iron recycling (12–15). Together, these effects of inflammatory mediators result in a functional iron deficiency, and erythropoietic cells are cut off their iron supply by macrophages. Presumably, this iron-storing macrophage phenotype deprives infectious agents as well as malignant cells from circulating iron sources. However, in the tumor microenvironment (TME), tumor-associated macrophages (TAM) may lose their ability to store iron because they are re-programmed by neoplastic cells to resume iron export. The mechanisms that result in metabolic reprogramming of TAM are incompletely understood but may involve transcriptional regulations and epigenetic changes (16).

**Figure 2:**
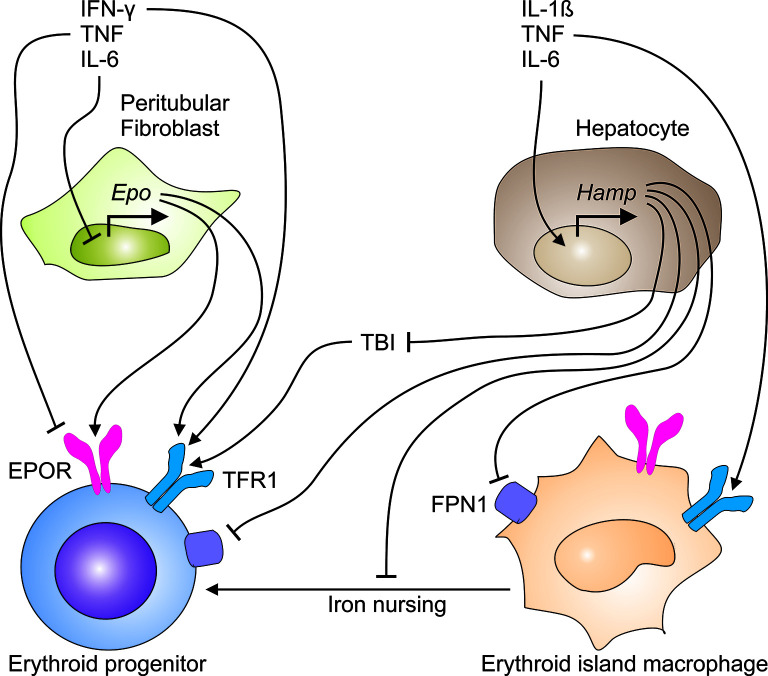
Immune-mediated pathways contribute to the development of cancer-induced anemia. The immune response to cancer cells generates a plethora of pro-inflammatory cytokines. TNF and other T_H_1 cytokines inhibit both, the production of erythropoietin (EPO) in peritubular fibroblasts in the kidney and the expression of the EPO receptor (EPOR) on erythroid progenitor cells in the bone marrow. In parallel, TNF, IL-1ß and IL-6 induce the transcriptional expression of the *hepcidin antimicrobial peptide* (*Hamp*) gene in hepatocytes. Liver-derived hepcidin acts on ferroportin (FPN)-1 and blocks its iron export function, thus reducing the transfer of iron to the circulation and the amount of transferrin-bound iron (TBI), consequently. In addition, hepcidin may undermine the transport of iron from erythroid island macrophages to erythroid progenitors, thus impairing their proliferation and hemoglobin synthesis.

Second, inflammatory mediators such as TNF and hydrogen peroxide inhibit the production of erythropoietin (EPO) in renal peritubular fibroblasts (**Figure 2**) (17, 18). Again, this mechanism aims at reducing the oxygen supply to tumor cells. However, these cells may switch to anaerobic glycolysis and induce tumor neovascularization by starting to generate angiogenetic factors. However, macrophages, endothelial cells or other cell types in the TME are also able to secrete angiogenetic mediators (19–21). Therefore, hypoxia in the TME is also a potential driving force for disease progression and metastasis (22). Although EPO levels are elevated in patients with CIA, this elevation remains insufficient for the degree of anemia (23–25).

Third, the immune response, intended to primarily target malignant cells, also impairs erythropoiesis in the bone marrow (**Figure 2**) due to inhibition of and damage to erythroid progenitors (EP) and hematopoietic stem cells (26–28).

Fourth, in advanced solid tumors, proliferating cancer cells may deprive EP of cobalamin and folate, infiltrate the bone marrow, displace normal hematopoiesis and destroy its niches similar to what is typical of hematologic malignancies. Without cancer treatment, all these mechanisms interact in a vicious circle to aggravate the severity of CIA. However, when cancer treatment is initiated, the toxic effects of chemotherapeutics or radiation therapy on the bone marrow and on other organs such as the kidney may contribute to anemia, too. Moreover, cellular iron retention counteracts the ability of hypoxia inducible factors (HIF) to stimulate EPO production, a mechanisms which is relevant to peritubular fibroblasts (29).

In summary, many of the major pathways that contribute to anemia in cancer patients interact with each other, and specific treatment for CIA needs to be taken into consideration to break this circle of mechanisms causing and aggravating conditions of anemia (30).

## Inflammation and Cancer Dysregulate Iron Metabolism

Iron metabolism and the immune response are interlinked (31). Iron is an essential nutrient to almost all microbes (32, 33). One important role of the acute phase response (APR) is the reduction of iron levels in extracellular compartments (i.e. the serum) and iron storage in the intracellular compartment (i.e. in FT). As for many other immune functions, this important role is fulfilled by monocytes, macrophages and other cellular players of the mononuclear phagocyte system (MPS). Macrophages possess a broad spectrum of pattern recognition and scavenger receptors for the sensing and uptake of potentially harmful macromolecules and the recognition of malignant cells. These receptors are linked to intracellular signaling cascades which converge at the level of key inflammatory transcription factors including the nuclear factor (NF)-κB (**Figure 1**). Once activated, NF-κB orchestrates the transcriptional responses of macrophages and other immune-competent cell types to *trans*-activate inflammatory gene products such as TNF, IL-6 and IL-22 which also play an important role in the cancer development and aggravation of CIA (34). These pro-inflammatory cytokines, especially the APR-initiator IL-6, target the liver and stimulate hepatocytes to increase hepcidin output (35–37). In inflammatory conditions such as in patients with malignancies, hepcidin expression is induced by the concerted interaction of two pathways involving IL-6, Janus kinases (JAK) and signal transducers and activators of transcription (STAT) or activin B and activation of the SMAD (for sisters of mothers against decapentaplegic)-1/5/8 signaling pathway (38–42). On the contrary, patients treated with an anti-TNF antibody or an anti-IL-6 antibodies exhibit reduced levels of inflammatory markers such as IL-6, hepcidin, and/or C-reactive protein, correlating with improvement in anemia related to autoimmune inflammatory conditions (43).

## The Acute Phase Response Drives Iron Retention in Macrophages

The immune response to pathogen associated molecular patterns (PAMP) such as lipopolysaccharide and to danger associated molecular patterns (DAMP), present in the TME, such as free heme, adenosine, IL-1α, high-mobility group box-1 (HMGB1) and S100 proteins are similar. The recognition of either PAMP or DAMP results in the activation of NF-κB, p53, mitogen-activated (MAP) kinases and inflammasomes (44). As a consequence, the hypoferremia of the APR also limits the availability of iron for malignant cells. Yet, prolonged hypoferremia may be regarded as a maladaptation to persistent immune stimulation in the setting of chronic infections, autoimmune or neoplastic diseases.

T cells are major mediators of the immune response against malignant cells. Specifically, major histo-compatibility (MHC) class I molecules present on tumor cells present neoantigens on their surfaces. These neoantigens are detected by cytotoxic T cells and elicit the secretion of granzyme B, perforins and of cytokines such as TNF and IL-6. Both cytokines induce hepcidin, a hormone with a unique mode of action: hepcidin binds to its receptor ferroportin (FPN)-1, an iron export channel, and blocks its transport function. Hepcidin thus mediates a negative feedback loop because it reduces iron recycling by macrophages and iron absorption by enterocytes when serum and tissue iron levels are elevated. Therefore, excess hepcidin deactivates iron transport to the blood stream resulting in a reduction of serum iron concentrations during ongoing iron consumption by transferrin receptor (TFR)-1 expressing cells in the face of reduced resupply (**Figure 2**).

To further support the iron withdrawal from malignant cells, IL-6, IL-10 and other pathways induce the iron storage protein FT (45, 46). FT is composed of 24 subunits of heavy and light chains which assemble in variable proportions to form a shell-shaped heteromultimer. Serum FT is primarily produced and secreted by macrophages (47). Therefore, the levels of serum FT reflect body iron stores and the state of immune activation. In other words, in the presence of cancer cells or other immune stimuli, serum FT does not accurately predict the amount of iron stored in macrophages and other cell types such as hepatocytes. Consequently, in these scenarios, normal serum FT does not rule out the depletion of iron stores that characterizes absolute iron deficiency. *Vice versa*, increased serum iron (hyperferritinemia) can indicate either parenchymal iron overload or immune activation with subsequent uptake and storage of iron in macrophages. In clinical settings, it is fundamental to distinguish the different etiologies, as treatment is directed toward the underlying morbidity and does not automatically result in iron reduction approaches.

## Iron Has Pleiotropic Effects on the Immune Response

**Figure 3:**
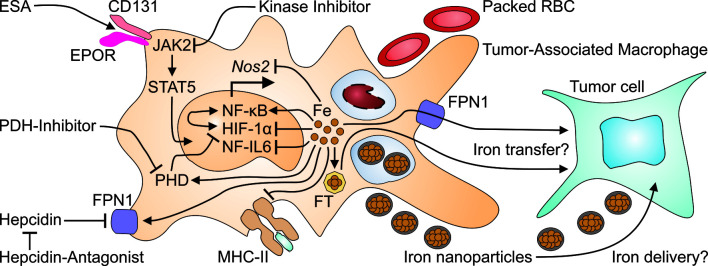
Many therapeutics for cancer-induced anemia may exert off-target effects on tumor-associated macrophages. Erythropoiesis-stimulating agents (ESA) activate the EPOR/CD131 heterodimer to initiate signaling *via* Janus kinase (JAK)-2 and signal transducer and activator of transcription (STAT)-5. On the other hand, kinase inhibitors, prescribed to treat cancer or to reduce hepcidin production, may interfere with JAK2 signaling. Packed red blood cells (RBC) are taken up by macrophages including TAM at the end of their life span and increase intramacrophage iron levels. Intravenous iron preparations are nanoparticles which are taken up by TAM by phagocytosis and increase the cellular iron content. Surplus intracellular iron in turn, can inhibit the transcriptional expression of the *nitric oxide synthase* (*Nos*)-*2* and of *major histocompatibility* (*MHC*) *class II* unless it is exported through FPN1 or stored in ferritin (FT). Presumably, either form of iron may be transferred from TAM to adjacent tumor cells. This may also be relevant for the application of hepcidin antagonists which prevent the action of hepcidin on FPN1, thus restoring macrophage iron export. Intracellular iron also has profound effects on TAM themselves because iron stimulates the binding activity of nuclear factor (NF)-κB, while reducing the activation of NF-IL6 and of hypoxia-inducible factor (HIF)-1α. The latter effect is also relevant to treatment with prolyl hydroxylase domain (PHD) inhibitors which impair the degradation of HIF-1α, much like iron deficiency which stabilizes HIF-1α's active form.

The adaptation of iron metabolism during the APR may have evolved to deal with acute stressors such as bacterial infections. However, when inflammatory stimuli persist, as is the case in neoplasms that cannot be fully resected, this immune-mediated storage of iron in the MPS may be of disadvantage for the affected individual for at least two reasons. First, iron storage in the MPS is the basis for a functional iron deficiency in the erythron. Second, while iron promotes the non-enzymatic generation of reactive oxygen species (ROS), it has negative effects on many other immune effector pathways. Macrophage iron overload, commonly resulting from chronic hemolysis and/or repetitive red blood cell (RBC) transfusions, impairs their effector functions which are promoted by TNF and interferon (IFN)-γ. These key cytokines are produced by cytotoxic T cells, T helper type 1 (T_H_1) cells and natural killer (NK) cells. Specifically, an increased macrophage iron content results in impaired production of nitric oxide (NO). This is a consequence of iron inhibiting the abilities of HIF-1 and of NF-IL6 to *trans*-activate the *NOS2* (for NO synthase-2) gene (**Figure 3**) (48, 49). Similarly, iron impairs MHC class II expression in macrophages (50). The transcriptional mechanisms are unknown yet. Therefore, iron may inhibit most T_H_ cell responses. In a similar fashion, surplus iron can also be directly toxic to T cells and inhibit their proliferation or induce ferroptosis (Tymoszuk et al., 2020). The latter is a specific form of cell death that is dependent on iron, ROS and lipid peroxides. Ferroptosis is mediated by inactivation of the lipid repair enzyme glutathione peroxidase 4 (GPX4) (51). In macrophages, the induction of ferroptosis results in degradation of FT. This process is known as ferritinophagy. Another effect of ferroptosis is the release of iron by nuclear receptor coactivator (NCOA)-4-mediated autophagy. As ferroptosis is an increasingly recognized mechanism of action of chemotherapeutics, the pathway provides another possible mechanism of interaction between iron metabolism, anti-tumor immunity and cancer biology (52–54).

In conclusion, iron has multiple, predominately negative effects on the immune response.

## Some Macrophage Populations Professionally Handle Iron

Macrophages are dispersed throughout the human body to assume organ- and tissue-specific forms and functions. Several macrophage populations exist for example in the spleen. Of major importance for the maintenance of body iron homeostasis are SpiC- and FPN1-expressing iron-recycling red pulp macrophages (RPM) (55, 56). Under steady-state conditions, RBC have a normal life span of 120 days. Finally, they display altered molecular surface patterns and begin to lose ‘don’t eat me’ signals. Moreover, RBC start to flip phosphatidylserine to their outer surface (57–59). RPM respond to these alterations and take up these RBC marked as ‘aged’ by a process known as erythrophagocytosis. In brief, engulfed RBC are degraded and their heme-iron is recycled to the systemic circulation by FPN1-mediated iron export (**Figure 3**). Alternatively, a novel study suggests that RBC may undergo physiologic hemolysis in the spleen and that their remnants are taken up by RPM for rapid turn-over (60). In situations when this capacity of RPM to take up damaged RBC (dRBC) and to recycle iron is overwhelmed, such as in massive hemolysis, Kupffer cells (KC) in the liver take over RBC uptake and degradation as a back-up mechanism. In addition, the liver-derived chemokines CCL2 and CCL5 stimulate the bone marrow to release new monocytes (61). These will then also participate in RBC uptake and iron recycling as a support to RPM and KC. Apart of these iron homeostatic functions, KC may play a role in tumor progression, too. *Per se* the liver is one of the organs most commonly affected by metastasis. Therefore, resident KC may be considered as TAM analogues for metastatic cells that have reached the liver. In this setting, iron-recycling by KC may facilitate the growth of liver metastasis. Similarly, in hepatocellular carcinoma, KC may be re-programmed to supply cancer cells with iron, resulting in adverse outcome (62, 63).

In summary, KC are a paradigm for macrophage populations that recycle iron and deliver this nutrient to cancer cells.

## TAM Are Macrophages in the TME

TAM are located in the TME and thus have a strategic position in immunity against malignant cells. However, tumor cells enter a cross-talk with TAM by producing soluble mediators and metabolites with which they can manipulate anti-tumor immune responses. Iron is a decisive factor in this interaction: The immune-mediated uptake of Hb by and the sequestration of iron in TAM both aim at withholding this nutrient from malignant cells to counteract disease progression (64, 65). *Vice versa*, tumor cells seek at undermining these mechanisms and may promote the release of iron from TAM (66). FPN1-mediated export of ionic iron and the secretion of iron-laden FT or lipocalin-2 may be the most relevant pathways by which re-programmed TAM supply neoplastic cells with iron (67, 68). In addition, malignant cells themselves produce lipocalin-2 and its receptor for the uptake of siderophore-bound iron (SBI) which supports cancer cell growth (69, 70). Moreover, tumor cells can express ionic iron importers such as divalent metal transporter (DMT)-1 and solute carrier family 39 member 14 (SLC39A14; also known as ZIP14) as well as receptors for FT and transferrin-bound iron (TBI) (71–73).

By producing the vascular endothelial growth factor (VEGF), TAM may also be implicated in tumor neovascularization which is triggered by tissue hypoxia, influenced by cardiovascular function and CIA, respectively (74). Hypoxia is sensed by oxygen sensitive prolyl hydroxylases (PHD) which stabilize HIF, a heterodimeric transcription factor composed of α and ß subunits. Under normoxic conditions, PHD continuously hydroxylate HIF-1α at two specific proline residues. This enzymatic process is modified by concentrations of ferrous iron and 2-oxoglutarate and tags HIF-1α for proteosomal degradation following binding by the von Hippel Lindau (VHL) E3 ubiquitin ligase complex. During cellular hypoxia, which commonly occurs in the TME, HIF-1α is stabilized thus enabling *trans*-activation of HIF target genes such as VEGF. VEGF also constitutes a relevant target for monoclonal antibodies, which are used as add-ons in combination with chemotherapeutics to counteract disease progression. The cross-talk between TAM and cancer cells is bidirectional, though. On the one hand, cells release lactate in breast cancer in order to stimulate TAM to transfer a HIF-1α stabilizing RNA into malignant cells. This impairs the action of chemotherapy (75).

On the other hand, PDH inhibitors, which are of potential use to treat CIA, may exert effects on TAM: PDH3 and HIF-1α play central roles in the activation of macrophages and in their interaction with T cells (76, 77). In addition, the central transcription factors HIF-1α and NF-κB are linked and put the PDH-HIF pathway right at the interface between the sensing of reduced oxygen and iron levels and immune effector functions (78, 79). Despite the key role of HIF-1α in TAM however (**Figure 3**), it is hard to predict whether these immunologic effects or potential pharmacologic intervention will favor or impair the progression of the underlying malignancy. In line, we currently lack a comprehensive understanding, how compounds for the treatment of CIA may affect the pleiotropic functions of TAM. In addition, many studies on the cross-regulation of iron metabolism and macrophage function address the functions of inflammatory cytokines and infectious agents but still need to be carefully translated into cancer models and, subsequently, cancer patients.

## TAM as Potential Targets of CIA-Directed Therapies

Macrophages form the central executive part of the MPS and are equipped with a plethora of pattern recognition and scavenger receptors. In order to fulfill their immune and homeostatic functions in the human body, macrophages need to respond to endocrine, paracrine and metabolic signals. The corresponding receptors present on macrophages include – but are not limited to – bone morphogenic protein receptor (BMPR)-I and -RII, hemojuvelin (HJV), transferrin receptor (TFR)-2, FPN1, IL6R, TNFRI, EPOR and CD (for cluster of differentiation)-131. Therefore, many therapeutic options for CIA are predicted to affect macrophage functions in general and TAM functions, specifically (**Figure 3**).

## Intravenous Iron Preparations Target Myeloid Cells

Intravenous iron preparations can be administered to cancer patients for two indications. First, patients with CIA and relevant absolute iron deficiency may benefit from intravenous iron supplementation as long as the underlying malignancy is under therapeutic control as well. Second, superparamagnetic iron oxide nanoparticles (SPION) are used for diagnostic purposes in imaging studies. Both classes of intravenous iron preparations are nanoparticles which primarily target classical monocytes and macrophages. Concretely, classical monocytes and macrophages take up iron-containing nanoparticles *via* their set of scavenger receptors and degrade them in phagolysosomes (80, 81). As a consequence, these cells accumulate their total and cytoplasmatic iron content before they transfer iron to the circulation (82). However, it is increasingly recognized that iron-containing nanoparticles exert immune-modulation on macrophages (83). For example, the clinically used intravenous iron preparations iron sucrose and sodium ferric gluconate impair the adhesion and phagocytosis of monocytes, while ferric carboxymaltose and iron isomaltoside lack these effects (84). However, also the latter two compounds affect the MPS. This is also evident from the fact that monocytes and macrophages take up iron carboxymaltose and deliver these iron-nanoparticles to tumors. There, they inhibit monocyte chemoattractant protein (MCP)-1 and to a lesser degree NO production (85). In contrast, the uptake of iron sucrose by circulating monocytes results in activation of NF-κB and in enhanced production of TNF, IL-6 and IL-8 (86, 87).

The uptake of iron nanoparticles by monocytes and macrophages is also relevant for the use of SPION as contrast enhancers for magnetic resonance imaging. SPION coated with dextran are phagocytozed by human monocytes, in which they activate mitogen-activated protein (MAP) kinases and stimulate TNF and IL-1ß production (88). Other SPION preparations however, counteract toll-like receptor (TLR)-4 signaling and NF-κB activation, thus reducing TNF, IL-1ß and IL-6 production (89). Further results obtained with monocyte-derived dendritic cells suggest that SPION impair antigen processing and T_H_ cell stimulation which may have important implications for anti-tumor immunity (90). Furthermore, efforts have been made to selectively label distinct macrophage phenotypes with SPION, which may be relevant for cancer patients because TAM represent distinct macrophage phenotypes (91).

In the face of these heterogenous data, it is currently difficult or even impossible to predict the clinical implications of the use of iron nanoparticles from their specific effects on TAM function. Therefore, more preclinical studies and following clinical data are desirable to better understand the effects of intravenous iron preparations on CIA and cancer.

## Blocking IL-6 or BMP-6 to Inhibit Hepcidin in CIA

The inhibition of hepcidin is an attractive treatment strategy for CIA patients, too. Hepcidin antagonism can be achieved *via* multiple approaches such as inhibitors of hepcidin expression, HJV inhibitors, hepcidin-binding oligoribonucleotides, neutralizing antibodies to hepcidin or antibodies which block its interaction with FPN1 (92, 93).

Tocilizumab, a monoclonal antibody approved for several indications such as rheumatoid arthritis, giant cell vasculitis or cytokine release syndrome, targets the IL-6R and thus one of the major pathways to induce the transcription of the *HAMP* (for hepcidin-antimicrobial peptide) gene (37, 94, 95). Therefore, tocolizumab ameliorates disease activity and lowers circulating hepcidin levels in rheumatologic diseases (96, 97). The latter effect improves ACD, presumably by a direct effect on hepatocytes (98). Nevertheless, one might speculate that blockade of IL-6R may also modulated the function of macrophages including TAM (99). For example, in macrophage activation syndromes, tocilizumab results in lower serum FT levels (100). The ligand IL-6 is also involved in the cross-talk between TAM and tumor cells and promotes the survival of tumor cells in hypoxic conditions and the differentiation of cancer stem cells (101–104). Whether or not this is relevant for the clinical application of tocilizumab in cancer patients remains to be addressed in further clinical trials. However, a plethora of studies has implicated chronic inflammation in general and IL-6, specifically, in tumor initiation and progression (105, 106). Therefore, IL-6R blockade may impair the progression and metastasis of some forms of cancer (107). Yet, these data do not exclude the possibility that tocilizumab facilitates malignant transformation in other malignant diseases (108). Thus, only clinical trials in cancer patients will give relevant answers as to whether or not tocilizumab is a safe and efficient therapy for CIA.

Other potential targets for the treatment of CIA are bone morphogenetic proteins (BMP) and their receptors. As members of the TGF-ß superfamily of cytokines, BMP-2 and BMP-6, are specifically involved in the maintenance of iron homeostasis, yet also immune regulation and cancer progression (41, 109). BMP-6 is secreted by endothelial cells in the liver to inform adjacent hepatocytes about elevated serum iron levels and replenished iron stores (110). Accordingly, BMP-6 is a major inducer of *HAMP* transcription and a potential mediator of CIA (111–113). Given the key role of BMP-6 for the induction of hepcidin, it is not surprising that neutralizing antibodies have been used to block hepcidin induction in preclinical and clinical models of ACD (114–116). As many cell types including normal and neoplastic epithelial cells as well as macrophages possess BMPR-I and -II, BMP-6 can affect both tumor cells and TAM.

Several studies have implicated BMP-6 in the linkage of immune regulation and cancer progression. In a mouse model of malignant melanoma for example, the absence of BMP-6 resulted in a substantial delay in tumor onset and progression by a mechanism depending on mast cells (117). In non-small cell lung cancer however, reduced BMP-6 expression was associated with reduced overall survival and BMP-6 inhibited the proliferation of lung cancer cells (118). Similarly, BMP-6 inhibited the growth of breast cancer cells induced by estrogens (119). A high expression of BMP-6 was associated with higher immune cell infiltration and better survival in estrogen receptor-positive breast cancer in a cohort study (120). In prostate cancer, BMP-6 produced by neoplastic cells acts on adjacent macrophages and activates NF-κB and SMAD1 signaling to increase IL-1α and IL-6 secretion. IL-1α, in turn, stimulates endothelial cells and promotes tumor angiogenesis (121). In parallel, IL-6 acts on BMP-6 secreting malignant cells and enhances the expression of the androgen receptor, a major determinant of tumor growth and treatment response in prostate cancer (122). In renal cell carcinoma (RCC), BMP-6 mediates a crosstalk between tumor cells and TAM, too. BMP-6 instructs TAM to assume an anti-inflammatory phenotype with increased IL-10 production. The expression of BMP-6 in RCC cell lines compared to that in a nonmalignant renal cell line correlated with RCC cell line proliferation Increased IL-10 levels predicted poor prognosis of RCC in human subjects (123, 124).

Given the pleiotropic effects of BMP-6 in the crosstalk between immune and neoplastic cells in the TME, it will be important to assess the effects of BMP-6 neutralizing antibodies on the clinical course of the underlying malignancies. In light of the available data, it is tempting to speculate that the gene signatures in biopsies from primary lesions and the role played by BMP-6 in a given tumor entity may enable us to predict whether BMP-6 blockade exerts stimulatory or inhibitory effects on tumor growth in an individual patient.

## The Hepcidin-FPN1 Axis Itself Is a Pharmacologic Target

Located downstream of IL-6 and BMP-6, the hepcidin-FPN1 axis itself is an attractive target for the therapy of CIA and other forms of ACD (125, 126). However, both FPN1 and its ligand hepcidin are potential regulators of tumor growth and the immune response directed against it. Loss-of-function mutations in *SLC40A1*, the gene encoding for FPN1, are in discussion to produce a molecule that does not traffic appropriately to the cell surface or that has limited ability to export iron (127–129). Excess accumulation of iron in macrophages is the result with accompanying high serum FT levels. In gain-of-function mutations of *SLC40A1*, the binding site of hepcidin is altered, resulting in a hepcidin-resistant protein and in iron overload (130–132). In TAM in RCC, FPN1 expression is elevated, especially in high grade lesions. Importantly, high FPN1 levels predict poor overall survival because iron export by TAM supports the proliferation and migration of RCC cells (133). Similarly, in breast cancer biopsies taken from the primary lesion and axillary lymph node metastases, TAM exhibit high FPN1 expression and cancer cells display high TFR1 levels, suggesting that TAM supply tumor cells with iron (134). Importantly, the influence that FPN1 levels on TAM exert on disease outcome, extend to other relevant malignancies including hepatocellular carcinoma (135). This may be a general observation for cancers with predominant infiltration by monocytes and macrophages. However, this effect may also partly be attributable to the role of FPN1 on macrophages in the control of their cellular iron status and immune response. Specifically, macrophages lacking FPN1 are impaired in their function and secrete higher amounts of TNF and IL-6, possibly because intracellular iron can stimulate the translation of these cytokines (136–138). On the other hand, over-expression of FPN1 in macrophages enhances their NO output because low intramacrophage iron levels promote the transcription of the *NOS2* gene (139, 140). FPN1 inhibitors are being evaluated in clinical trials for the treatment of thalassemia (141). However, to date, no data exist on the use of allosteric FPN1 modulators for ACD or CIA.

In contrast, humanized monoclonal antibodies which display a high affinity toward hepcidin and lead to its premature degradation have been developed. One of these, LY2787106, was shown to be tolerated well during its phase one clinical trial, demonstrating a significant increase in serum iron levels (142). Short hairpin RNA (shRNA) which target hepcidin were demonstrated to cause a reduction in hepcidin production and alleviate anemia when used in conjunction with erythropoiesis stimulating agents (ESA) because they may inhibit hepcidin more robustly than anti-hepcidin antibodies (143). Similarly, aptamers, an emerging class of synthetic, structured oligonucleotide therapeutics, can inhibit *HAMP* expression with high affinity and specificity, thus increasing iron availability for erythropoiesis in a preclinical ACD model (144, 145).

In conclusion, several approaches targeting the hepcidin-FPN1 axis may be effective in CIA because they have the potential to improve anemia in preclinical models of ACD (93, 146, 147). Not only does hepcidin target macrophages to limit iron recycling, it is also produced by macrophages themselves, possibly to autoregulate their iron homeostasis (148–150). Therefore, it is feasible to assume that hepcidin-targeting therapies will affect the immune functions of macrophage populations including TAM, raising safety concerns.

## The Hepcidin-FPN1 Interaction in Malignancies

The functional importance of the hepcidin-FPN1 interaction is not limited to TAM, though. In cancer cells, up-regulation of iron uptake pathways such as DMT1 or TFR1 and down-regulation of FPN1 keep cellular iron levels high for metabolism and proliferation (151–153). Also, low FPN1 expression in malignant cells has been linked to the proliferation of malignant myeloma (154, 155). It therefore comes as a surprise that low FPN1 levels are associated with improved prognosis in acute myeloid leukemia (156). However, this observation has been linked to increased sensitivity to chemotherapy, and it is currently being investigated whether iron-induced cytotoxicity or ferroptosis are contributing mechanisms (157).

As for solid tumors, the most conclusive data are available for breast cancer (134). In this tumor entity, several mechanisms cooperate to reduce FPN1 expression on tumor cells including epigenetic modifications in the *FPN1* promoter region and down-regulation of FPN1 protein by hepcidin which is secreted by cancer cells and adjacent fibroblasts (158–160). Of relevance, expression levels of hepcidin and FPN1 govern disease outcome in breast cancer and decreased *SCL40A1* gene expression is an independent predictor of reduced metastasis-free and disease-specific survival (161).

**Figure 4:**
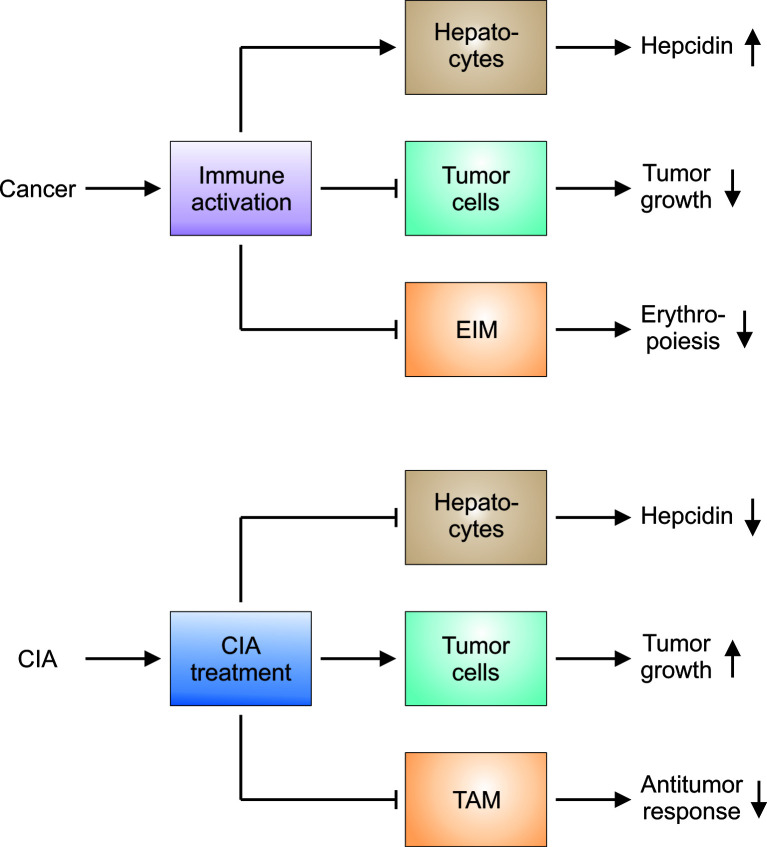
Hypotheses on the pathophysiology of CIA and the effects of CIA treatment on the underlying tumor. Upper panel: Cancer results in prolonged immune activation, especially when the disease is advanced or progressive. The immune response directed against malignant cells impairs tumor growth. At the same time, proinflammatory mediators stimulate hepatocytes to produce hepcidin: Hepcidin in turn counteracts iron export by erythroid island macrophages (EIM). Other immune mechanisms exert similar effects on EIM and aggravate the functional iron deficiency observed in cancer patients. Thus, several mechanisms contribute to the development of cancer-induced anemia (CIA). Lower panel: CIA and the underlying functional iron deficiency can be considered maladaptations to prolonged immune activation. CIA treatment is intended to counteract these immune-mediated pathways and to promote erythropoiesis. Many of these therapeutic approaches reduce hepcidin production or block its negative action on ferroportin-mediated iron export. However, CIA treatment may exert unintended side effects on tumor cells and on tumor-associated macrophages (TAM). The latter may be attributable to the fact that EIM cannot be selectively targeted and other macrophage populations such as TAM are inhibited by CIA treatment, too. Therefore, all therapeutic interventions for CIA have to be thoroughly tested in preclinical models and clinical trials to check for unintended effects on tumor growth and the antitumor response.

In conclusion, there is overwhelming evidence that the hepcidin-FPN1 axis regulates both, the function of TAM and the growth of cancer cells. While antagonizing the hepcidin-FPN1 interaction may ameliorate CIA because one of the key molecular mechanisms of functional iron deficiency is undermined, there may be relevant further consequences for the underlying disease (**Figure 4**). Blocking the hepcidin-FPN1 interaction on TAM will reduce their iron content and may promote NO production, MHC class II expression and alter cytokine production, consecutively increasing their anti-tumor activities. In contrast, FPN1 mediated iron export from TAM may increase the availability of iron not only for EP, as intended, but also for cancer cells. On the other hand, the neutralization of hepcidin’s effects on FPN1 present on cancer cells may inhibit their proliferation yet impair ROS-mediated effects of chemotherapeutics. Therefore, any compound that targets the hepcidin-FPN1 axis exerts both systemic and local effects and needs to be thoroughly tested in preclinical cancer models and clinical cancer trials to carefully balance the benefit-risk ratio of such products before approval for clinical use.

## Diagnosis of CIA

### Individual Approach for Cancer Patients

CIA is a subtype of ACD and may be viewed as spectrum by itself that ranges from mild and asymptomatic forms in individuals with well-controlled residual malignancy to severe forms that, if left uncorrected, will limit patients’ life expectancy just as does the underlying neoplasia (162). Anemia in cancer patients can be classified as CIA when the anemia has hyporegenerative features as indicated by a reduced reticulocyte count or reticulocyte production index (163). Often, the underlying cancer is advanced or not in remission or patients are under chemotherapy (164). In many cases of CIA however, substantial hemolysis, evident from haptoglobin consumption, contributes to anemia as do other contributing factors such as EPO and vitamin deficiencies. Given this multifactorial etiology of CIA, cancer patients often require an individual approach in the diagnosis and treatment.

### CIA and Laboratory Markers

CIA is often normocytic or microcytic but occasionally macrocytic. Because of the fact that serum FT is elevated as part of the APR, a higher cut-off of 100 rather than 30 ng/ml for the assessment of body iron stores in cancer patients may be necessary (165). In cancer patients, serum FT < 100 ng/ml and TSAT < 20% suggest absolute iron deficiency but the Hb content of reticulocytes may be a more reliable parameter (166). Similarly, the soluble TFR is less affected by inflammation and may thus be a reliable indicator of absolute iron deficiency, even in the setting of cancer (167). This may be attributable to the idea that the Hb content of reticulocytes is a marker of the functional ID whereas the FT index (soluble TFR/log FT) primarily reflects the iron availability for erythropoiesis (168, 169). Therefore, the FT index facilitates the differential diagnosis between iron deficiency and ACD (170). An sTFR-FT index <1 suggests ACD, whereas, an sTFR-FT index >2 suggests ACD accompanied by absolute iron deficiency anemia. The use of the FT index for daily clinical routine is limited by the lack of internationally standardized assays, though (171).

### BMP-6 and Hepcidin Elevation in CIA

BMP-6 and hepcidin levels are elevated in CIA which may aid in the diagnosis (112, 113). Yet it remains unclear whether serum concentrations of these mediators predict the response to currently available treatments such as ESA and to therapies specifically targeting them (172). Also, hepcidin levels may be regulated by cancer-induced inflammation through IL-6 dependent and independent pathways (167, 172, 173).

In conclusion, both patient’s history as well as classical and novel laboratory parameters enable the accurate classification of CIA. Given the increasing number of parameters available, we expect that improved algorithms for the differential diagnosis of CIA and the prediction of treatment responses will become available in the near future.

## Treatment Indications and Options for CIA

### Aim of CIA Treatment

All inner organs depend on a sufficient supply with oxygen from its iron-containing carrier Hb. CIA thus impairs organ functions and activities of daily living in patients with neoplastic disorders. In addition, CIA may negatively affect the survival of cancer patients (174, 175). Therefore, a correction of the reduced Hb levels is warranted although the desired target levels remain incompletely studied and thus still under debate.

### Different Treatment Options in CIA

The therapeutic options to correct the CIA have increased in recent years (**Table 1**). Apart of RBC transfusions and intravenous iron preparations, ESA have long been used to treat CIA. Recently, hepcidin antagonists such as monoclonal antibodies, short interfering RNA (siRNA), shRNA, aptamers, TFR2 inhibitors as well as pharmacologic inhibitors of HIF prolyl hydroxylases, e.g. vadadustat, roxadustat and daproustat, have emerged as novel therapeutic concepts for CIA. RBC transfusions and ESA are described more in detail as cornerstones of CIA treatment in the further sections.

**Table 1:** Selected approved and experimental treatment options for CIA.

Compound/group	Target/mechanism	Pharmacological/clinical effect
Intravenous iron preparations	Deliver iron-containing nanoparticles to macrophages	Correction of absolute iron deficiency
Tocilizumab	Blocks the IL-6 receptor	Suppresses IL-6 induced immune pathways including hepcidin production
LY2787106	Neutralizes circulating hepcidin	Blocks hepcidin and restores iron transfer to the circulation *via* FPN1
KY1070	Neutralizes circulating BMP-6	Reduces hepcidin transcription and restores iron transfer to the circulation *via* FPN1
Momelotinib	Off-target inhibition of BMPR kinase activin A receptor, type I	Reduces hepcidin transcription and restores iron transfer to the circulation *via* FPN1
TFR2 inhibitors	Inhibit TFR2 on erythroid cells	Improve EPO sensitivity
ESA	Stimulate the EPOR on erythroid progenitor cells	Restore erythropoiesis
HIF prolyl hydroxylase inhibitors	Stabilize HIF	Restore endogenous EPO production
RBC	Transfusion of allogenic RBC	Delivery of RBC as oxygen carriers

## Red Blood Cell Transfusions

RBC transfusions have long been used to correct anemia, including CIA. The direct replacement of RBC and rise in Hb levels promptly ameliorates oxygen supply to vital organs including the central nervous system and myocardium and thus promotes quality of life and exercise capacity. Yet, this strategy can also worsen the underlying malignancy because oxygen delivery to neoplastic cells will increase, too. Furthermore, RBC transfusions may also modulate the immune response in recipients because packed RBC contain up to 0.8% of hemolyzed cells (176). Therefore, during transfusion, substantial amounts of free Hb, heme and iron as well as microvesicles and membrane fragments can enter the circulation. To avoid tissue damage and inflammation, strategically located macrophage populations such as RPM in the spleen and KC in the liver will neutralize these compounds by CD163, CD91 and other scavenger receptors. CD163 recognizes both free and haptoglobin-bound Hb and eliminates it by receptor-mediated endocytosis. CD91, on the other hand, binds heme-hemopexin complexes. Subsequently, intracellular heme is degraded by heme oxygenase-1 (HO1) and detoxified to bilirubin. When HO1’s enzymatic capacity is overwhelmed, and heme starts to accumulate within cells, free heme activates the NLRP3 inflammasome (177). Similarly, in the extracellular space, macrophages sense free heme as DAMP and initiate the APR (178). Therefore, by capturing free Hb and free heme, the CD163 and CD91 pathways protect from pro-oxidative tissue damage at the systemic level. In the TME, however, their functions may be different because CD163 is a marker of TAM and may enhance the delivery of iron to the tumor (179).

In clinical practice, the major advantage of RBC transfusions is the rapid improvement of Hb levels, which must be balanced against the risks for immune-mediated adverse reactions and transmissible infections.

## Erythropoiesis-Stimulating Agents for CIA Treatment

### EPO Functions and Signaling in CIA

In general, ESA are derivatives of the endogenous hormone EPO. EPO itself has a dual function in human biology: On the one hand, it is the key growth factor for EP in the bone marrow. There, EPO activates its homodimeric receptor to promote the differentiation of multipotent hematopoietic progenitors along the erythroid lineage and inhibit the apoptotic elimination of surplus cells (**Figure 2**) (180). On the other hand, EPO exerts functions of an anti-inflammatory and tissue-protective cytokine throughout the body. These latter functions are mediated by a distinct molecular form of its receptor, a heteroreceptor of EPOR and CD131 (181). This extraerythropoietic receptor is also expressed by immune cells including T cells and macrophages (**Figure 3**). EPO’s effects on the immune system may be either pro- or anti-inflammatory dependent on the type of EPO-responsive cells, the context of the tissue microenvironment or the entity of underlying disease. In CIA, ESA raise Hb levels and reduce the frequency of RBC transfusion in patients.

### Cell-, Organ- and Malignancy-Specificities of EPO

In the liver, EPO enhances the phagocytotic capacity of KC and the production of CCL2. This chemokine, in turn, promotes the recruitment of monocytes from the bone marrow to the liver, but it is also implicated in tumor metastasis (182–184). Also, in lung epithelial cells, EPOR expression is higher in malignant cells than in normal cell types (185). This suggests that unintended side effects of EPO are more likely to occur in CIA than in other forms of ACD. In malignant myeloma for instance, EPO stimulates IFN-γ production and counteracts disease progression. In bone marrow derived macrophages in contrast, EPO promotes the secretion of angiogenetic factors which may drive myeloma progression. This function may be especially relevant in the setting of multiple myeloma because in these patients, bone marrow macrophages overexpress EPOR (186). On the other hand, EPO may induce apoptosis in myeloma cells (187). This may be relevant for erythroid island macrophages, on which EPOR is highly expressed and may be important for their nursing function and for the delivery of iron to adjacent EP (188).

As EPO and ESA can act on both, TAM and cancer cells, these compounds may impact on the malignant disease underlying CIA. ESA in the presence of EPOR may promote angiogenesis, tumor growth, tumor cell survival, or resistance to treatment. However, it is impossible to predict the net effect of ESA therapy in a given tumor entity. In gastroesophageal cancer for example, ESA therapy initiated at a similar Hb cut-off of 11 g/dl, tended to have improved clinical outcome, implying that in this context, ESA is a valuable adjunct therapy (189). In breast cancer patients with CIA however, treatment with ESA may not affect overall survival yet increase the risk of venous thromboembolic events (190, 191).

### Benefit/Risk Considerations of ESA in CIA

Previous meta-analysis have suggested that ESA may be efficient and safe for the treatment of CIA (192). However, more recent work has led to opposite conclusions, questioning the safety of ESA and raising concerns about the increased risk for thromboembolic events and deaths in CIA patients receiving these compounds: A systematic review and meta-analysis of 6,769 cancer patients in 35 clinical trials exhibited an increased risk of thromboembolic events with recombinant human erythropoietin compared with controls (relative risk (RR), 1.67; 95% confidence interval (CI), 1.35 to 2.06) (193). A meta-analysis of patient-level data from 53 controlled trials in cancer patients who received chemotherapy, radiation therapy, chemoradiotherapy, or no therapy with epoetin therapy (n=13,933) reported a consistently significantly increased risk of thromboembolic events (194). The absolute event rates ranged from 0 to 30.8% (pooled 5.8%) in the treatment arms and from 0 to 14.5% (pooled 3.2%) in the control arms. Besides, other off-target effects of ESA might contribute to mechanisms of tumor regulation, such as cell activation and neovascularization. Based on the results on thromboembolic safety and mortality, ESA are not recommended for the treatment of anemia that is unrelated to chemotherapy in patients with malignancy. Potential exceptions of use of ESA are in patients with lower risk myelodysplastic syndromes to avoid RBC transfusions and the use in patients with concomitant renal failure. The main advantage of ESA is the decreased need for RBC transfusions.

To date, it remains unknown whether and how ESA affect tumor growth or their control by the immune system. From a clinical standpoint, in the absence of conclusive evidence, a personalized approach is mandatory in order to balance the potential benefits of ESA treatment, taking into account each patient’s individual circumstances and preferences, against the increased risk of thromboembolic events and death.

In summary, we need further preclinical and clinical research to characterize the cellular mechanisms and molecular pathways by which ESA affect thrombogenesis and tumor growth in patients with CIA (195).

## Conclusions

As for other forms of ACD, treating the underlying disease is the preferred therapeutic approach to patients with CIA. In patients with progressive disease however, it may be required to treat CIA *per se* in order to positively influence the cancer patient’s quality of life, physical performance and life expectancy.

Nowadays, physicians have an increasing armamentarium at hand to treat CIA. In patients, in whom the underlying malignancy is in full remission and in regular follow-up, treatment of CIA may be relatively safe and the erythropoietic bone marrow is likely to benefit from ESA, iron supplementation or hepcidin antagonism. In progressive cancers however, there may be an increased risk that CIA-directed interventions in fact provide malignant cells with growth promoting nutrients and/or signals. Therefore, a combination therapy that stimulates erythropoiesis with ESA on the one hand and provides EP with iron by medications targeting the IL-6R, BMP-6, hepcidin or FPN1 may be the preferred approach because in combination, these compounds may preferentially supply EP with signals and nutrients for proliferation.

In the future, we expect to have improved mathematical models and IT supported algorithms to diagnose and subclassify CIA, select multimodal therapies and predict treatment responses. We need to conduct appropriately powered randomized controlled trials in order to evaluate the benefits and risks of therapeutic interventions for CIA. Further objectives are trials with end points that focus on overall survival, disease free survival, progression free survival, exercise capacity, infection rate and quality of life. Before complementing these clinical studies, ongoing work in preclinical cancer models aims to gain further mechanistic insight in the effects that CIA-directed therapies exert both locally, on TAM and other tumor infiltrating leukocyte populations, as well as systemically (**Figure 4**).

Therefore, both preclinical and clinical investigations are inevitable to ameliorate – or TAM-e – CIA with acceptable risks of medicine.

## Data Availability Statement

The original contributions presented in the study are included in the article/supplementary material. Further inquiries can be directed to the corresponding author.

## Author Contributions

SW has written the manuscript. MN has written the manuscript and drawn the figures. All authors contributed to the article and approved the submitted version.

## Funding

Work in MN’s laboratory is supported by grants from the Austrian Research Fund (FWF stand-alone project P 33062) and the Tyrolean Research Fund (TWF).

## Disclaimer

SW is a member of the Pharmacovigilance Risk Assessment Committee (PRAC) of the EMA and the Human Medicines Expert Committee (HMEC) of Swissmedic. The views expressed in this article are the personal views of the authors and may not be understood or quoted as being made on behalf of or reflecting the position of an agency or one of the committees or working parties.

## Conflict of Interest

The authors declare that the research was conducted in the absence of any commercial or financial relationships that could be construed as a potential conflict of interest.
